# Sex-Specific Effects of Cumin Supplementation on Body Composition, Lipid Levels, and Glycemic Profiles: A Pilot Study

**DOI:** 10.7759/cureus.82774

**Published:** 2025-04-22

**Authors:** Shin Suzuki, Ayako Otsuka, Etsuko Kurata, Kimito Mio, Tasuku Inaba, Kentaro Yoshida, Shoji Kinoshita, Toyohiro Hamaguchi, Eun Sangsoo, Masahiro Abo

**Affiliations:** 1 Department of Rehabilitation Medicine, Jikei University School of Medicine, Tokyo, JPN; 2 Department of Nutrition, Koyama Rehabilitation Hospital, Shizuoka, JPN; 3 Department of Rehabilitation, Graduate School of Health Sciences, Saitama Prefectural University, Saitama, JPN; 4 Department of Rehabilitation Medicine, Koyama Rehabilitation Hospital, Shizuoka, JPN

**Keywords:** body composition, cumin, lipid profile, muscle quality, sex differences

## Abstract

Objectives: Cumin (*Cuminum cyminum *L.) is a medicinal plant in the Apiaceae family that is considered promising for treating lifestyle-related diseases such as obesity, diabetes, and dyslipidemia. However, sex differences in the effects of cumin have not been well studied. This study aims to clarify the effects of cumin on body composition and lipid and glucose profiles and examine the differences in these effects between men and women.

Design and methods: We conducted a before-and-after trial with 29 healthy adults aged 22-74 at a single facility. After a two-month pre-intervention period, participants consumed 2 g of cumin powder daily for two months. We measured body composition using bioelectrical impedance analysis. Additionally, we assessed blood biomarkers, including lipid and blood glucose profiles, at baseline and both before and after the intervention period. We analyzed the data using Student's t-tests and Wilcoxon rank-sum tests.

Results: In women, cumin intake significantly reduced the extracellular-to-total body water ratio and increased phase angle, suggesting improved muscle quality. Low-density lipoprotein cholesterol (LDL-C) decreased significantly in both sexes, while high-density lipoprotein cholesterol (HDL-C) decreased in men only. There were no significant changes in glucose profiles.

Conclusion: Cumin showed sex-specific effects on body composition and lipid profiles. Muscle quality improved in women only following cumin consumption, and while its effects on LDL-C were similar in both sexes, its effects on HDL-C were specific to men. These findings indicate that cumin's health benefits vary by sex, highlighting its potential for personalized use.

## Introduction

Cumin (*Cuminum cyminum* L.), a medicinal plant in the Apiaceae family, is an annual or biennial herb extensively cultivated in regions like the Middle East, India, China, and various Mediterranean countries, including Tunisia [1]. Cumin seeds are rich in volatile oils, essential fatty acids, proteins, and bioactive compounds such as cuminaldehyde and flavonoids, making them popular as a spice in numerous countries. They also contain thiamine, riboflavin, niacin, vitamins B6, C, and E, and minerals such as calcium, iron, magnesium, phosphorus, potassium, sodium, and zinc [2].

Cumin has been reported to have beneficial effects on human health, such as improving lipid profiles [3-6], blood glucose profiles [3,7], and obesity [5,6]. Two studies reported that a daily intake of 3 g of cumin for eight weeks in middle-aged women with dyslipidemia significantly increased paraoxonase 1, an antioxidant enzyme, and markedly reduced total cholesterol (TC), low-density lipoprotein cholesterol (LDL-C), and triglycerides (TG) [4,6]. In women with obesity, a similar intake led to significant reductions in TC, TG, and LDL-C but an increase in high-density lipoprotein cholesterol (HDL-C) [4,6]. These interventions, however, were limited to women, and no studies thus far have involved men. Differences in fatty acid oxidation between sexes and the influence of sex hormones on fatty acid oxidation suggest that responses to dietary interventions may vary between men and women, although no recent research has addressed how cumin consumption affects sugar and lipid metabolism in different sexes [8-12].

The beneficial effects of cumin for improving the blood glucose profile in diabetic patients have also been investigated. Keihan et al. reported significantly lower fasting blood glucose and hemoglobin A1c (HbA1c) levels in type 2 diabetic patients who consumed essential oil extracted from cumin relative to a control group [3]. The improvement in blood glucose profile is attributed to the pharmacological effects of cuminaldehyde, a component of the essential oil, which is believed to inhibit the α-glucosidase and aldose reductase enzymes involved in glucose metabolism [13]. Although each drug has been developed as a therapeutic agent for diabetes, the differences in efficacy between men and women have not been examined recently. Cumin contains numerous bioactive compounds, such as alkaloids, flavonoids, and terpenoids [14].

Flavonoids have recently been shown to potentially have positive effects on the musculoskeletal system, including improvements in muscle mass and muscle strength, as well as exerting protective effects against muscle atrophy [15,16]. Further, in recent years, attention has been drawn to evaluating muscle quality not only based on muscle mass but also by using the intracellular-to-extracellular water ratio (ECW/TBW) and phase angle [17-19]. Bioelectrical impedance analysis (BIA) is a method that measures whole-body impedance, which reflects the opposition of body tissues to an applied alternating electrical current. Impedance consists of two components: resistance (R), which represents the opposition to current flow through conductive tissues such as body fluids, and reactance (Xc), which reflects the capacitive properties of cell membranes. Phase angle is derived directly from these components and calculated as the arctangent of reactance to resistance, using the following formula:

$Phase angle (degrees) = arctangent (Xc / R) &times; 180&deg;/&pi;$

Higher phase angle values are considered indicative of greater cell density and better cellular membrane integrity and are therefore associated with greater quantity and quality of soft tissue [20,21]. It has been reported that a Mediterranean diet improves phase angle and that a healthy diet is associated with higher phase angle values, shedding light on the relationship between diet and muscle quality [22,23]. Although sex differences in muscle mass and muscle quality are well known, the impact of cumin intake on muscle and whether these effects differ by sex have not been studied thus far [24-26].

Based on these findings, we hypothesized that cumin might not only improve sugar and lipid profiles but also body composition, potentially with different outcomes between sexes. This pilot study was conducted to determine the effects of cumin powder on sugar and lipid profiles and body composition in both men and women.

## Materials and methods

Cumin

The cumin used in this study was a standardized product of Turkish origin, provided by S&B Foods, Inc. (Tokyo, Japan). Although the cumin used was a commercially standardized product, detailed quantification of its active compounds was not conducted.

Participants

This study was a pre-post comparison trial conducted at Koyama Rehabilitation Hospital in Shizuoka Prefecture, Japan, from August to November 2022. Adult employees of the same hospital were recruited for participation through an internal bulletin. The following exclusion criteria were applied: allergy to cumin or other plants in the Apiaceae family, diagnosis of any severe medical condition, use of medications that could affect glucose or lipid metabolism, or being pregnant or possibly pregnant. All 29 individuals who expressed interest met the eligibility criteria and were enrolled in the study.

Written informed consent was obtained from all participants prior to enrollment. The study was conducted in accordance with the guidelines outlined in the Declaration of Helsinki, and all procedures involving human participants were approved by the Ethics Committee of Koyama Rehabilitation Hospital (approval number: 2022-1). This study was registered in the University Hospital Medical Information Network (UMIN) Clinical Trials Registry (ID: UMIN 000057446).

Study design

This study was conducted as a single-arm, pre-post comparison trial without a separate control group, where each participant served as their own control. This study was conducted as a pre-post comparison trial. A two-month non-intervention observation period was followed by a two-month intervention period, during which participants consumed 2 g/day of cumin powder daily. Measurements were performed at three time points: (1) before the pre-observation period (baseline), (2) after the non-intervention period (pre), and (3) after the intervention period (post). No specific instructions were provided regarding the consumption (e.g., cooking or the time of day at which the cumin was taken), but the participants were instructed to avoid any changes in their exercise and dietary habits during the study period. Participants who consumed cumin powder less than five days per week during the intervention period were categorized as dropouts. Adherence to cumin intake was monitored through weekly self-reporting.

Body composition assessment

Body composition was assessed using a BIA device, the InBody S10 (InBody Co., Ltd., Seoul, South Korea). Measurements were taken after the participant had maintained a seated position for approximately 10 minutes in the morning following an overnight fast. The device measured fat mass, muscle mass, skeletal muscle mass index (SMI), ECW, TBW, intracellular-to-extracellular water ratio (ECW/TBW), and phase angle.

Blood sample analysis

Approximately 5 mL of venous blood was collected from the participants for biochemical analysis after fasting for at least 12 hours. Lipid profiles (TC, LDL-C, HDL-C, and TG) were measured using biochemical assay kits (Shinotest, Kanagawa, Japan). Analyses followed standardized protocols and were performed using an automated analyzer (Canon Medical Systems, Tochigi, Japan) at the laboratory of Koyama Rehabilitation Hospital. The internal and external coefficients of variation (CV) for these assays were all < 5%.

Blood glucose profiles (HbA1c and immunoreactive insulin (IRI)) were measured by a laboratory (BML General Research Institute, Saitama, Japan) after appropriate sample preparation. HbA1c was analyzed using the latex agglutination method (Minaris Medical, Tokyo, Japan), and IRI was measured via chemiluminescent immunoassay (CLIA) (Hitachi Chemical Diagnostics Systems, Tokyo, Japan). Using these values, we performed the homeostatic model assessment for insulin resistance (HOMA-IR) and calculated the quantitative insulin sensitivity check index (QUICKI).

Statistical analysis

All statistical analyses were performed using Python version 3.11.8 (Python Software Foundation, Fredericksburg, VA, USA) and its associated libraries, including Pandas, NumPy, and SciPy. The data were analyzed separately for men and women, as the primary objective was not to compare sexes but to explore sex-specific responses to cumin intake. For each sex, we performed repeated-measures ANOVA with age as a covariate to assess within-group changes across three time points (baseline, pre-intervention, and post-intervention).

When the assumption of normality was met (as verified by the Shapiro-Wilk test), we conducted pairwise comparisons using paired Student's t-tests. If the normality assumption was violated, we used the Wilcoxon signed-rank test instead. Although not all ANOVA results showed significant main effects, we proceeded with post-hoc comparisons to identify meaningful trends, given the exploratory nature of this pilot study. Effect sizes (Cohen's d or rank biserial correlation), 95% confidence intervals, and descriptive statistics were calculated and presented. Statistical significance was defined as p < 0.05. All results are presented as mean ± SE or median with interquartile range, depending on the data distribution.

## Results

All 29 participants (16 women and 13 men) successfully completed the two-month intervention of consuming 2 g of cumin powder daily. No adverse effects or events were reported by any participants during the study period. The participants' age, height, weight, and BMI are presented in Table 1, and their physiological parameters across the study period are shown in Table 2.

**Table 1:** Baseline Characteristics of Participants Consuming Cumin BMI, body mass index

Variable	Women (n = 16)	Men (n = 13)	Total (n = 29)
Age (years)	34 (23–63)	28 (22–74)	32 (22–74)
Height (cm)	157 (149–168)	170.5 (153–188)	161 (149–188)
Weight (kg)	53.25 (43–77.6)	63.6 (54.5–84)	60 (43–84)
BMI (kg/m^2^)	21.2 (18.5–33)	22.6 (18.1–27.7)	21.6 (18.1–33)

**Table 2:** Physiological Parameters by Gender and Test Time Point Test time points: baseline = before the pre-intervention period; pre = after the two-month pre-intervention period; post = after the two-month intervention period. ECW/TBW, extracellular-to-total body water ratio; HbA1c, hemoglobin A1c; HDL-C, high-density lipoprotein cholesterol; HOMA-IR, homeostatic model assessment for insulin resistance; IRI, immunoreactive insulin; LDL-C, low-density lipoprotein cholesterol; QUICKI, quantitative insulin sensitivity check index; SE, standard error; SMI, skeletal muscle mass index; TC, total cholesterol; TG, triglycerides

Variable	Women			Men		
	Test	Mean	SE	Test	Mean	SE
Fat mass	Baseline	16.30	2.12	Baseline	52.50	1.50
	Pre	16.20	2.12	Pre	53.00	1.71
	Post	16.00	2.16	Post	51.50	1.40
Muscle mass	Baseline	37.20	0.87	Baseline	10.80	1.62
	Pre	36.60	0.70	Pre	10.50	1.67
	Post	37.00	0.79	Post	12.50	1.54
SMI	Baseline	6.51	0.15	Baseline	8.16	0.13
	Pre	6.33	0.13	Pre	8.17	0.19
	Post	6.38	0.13	Post	7.93	0.12
ECW/TBW	Baseline	0.38	0.00	Baseline	0.38	0.00
	Pre	0.38	0.00	Pre	0.37	0.00
	Post	0.38	0.00	Post	0.37	0.00
Phase angle	Baseline	5.49	0.13	Baseline	6.62	0.19
	Pre	5.44	0.14	Pre	6.75	0.24
	Post	5.65	0.12	Post	6.55	0.17
LDL-C	Baseline	138.00	15.80	Baseline	123.00	7.54
	Pre	143.00	14.80	Pre	127.00	7.27
	Post	124.00	12.20	Post	110.00	6.96
HDL-C	Baseline	53.90	8.16	Baseline	44.70	5.32
	Pre	52.80	7.51	Pre	43.90	4.35
	Post	46.80	5.58	Post	37.80	2.94
TC	Baseline	208.38	12.745	Baseline	187.38	7.02
	Pre	213.69	12.556	Pre	193.15	8.54
	Post	213.38	11.891	Post	188.38	8.12
TG	Baseline	83.10	12.60	Baseline	82.80	9.77
	Pre	91.90	11.20	Pre	93.00	15.71
	Post	89.60	14.10	Post	95.50	12.08
HbA1c	Baseline	5.12	0.06	Baseline	5.17	0.06
	Pre	5.11	0.06	Pre	5.13	0.05
	Post	5.23	0.07	Post	5.10	0.07
IRI	Baseline	5.88	1.15	Baseline	4.65	0.68
	Pre	5.41	0.74	Pre	5.82	0.83
	Post	7.95	2.02	Post	5.34	0.66
HOMA-IR	Baseline	1.26	0.25	Baseline	1.03	0.16
	Pre	1.15	0.16	Pre	1.29	0.20
	Post	1.85	0.48	Post	1.17	0.16
QUICKI	Baseline	0.39	0.01	Baseline	0.40	0.01
	Pre	0.39	0.01	Pre	0.38	0.01
	Post	0.37	0.01	Post	0.38	0.01

Physiological and metabolic effects of the intervention in women

A repeated-measures ANOVA, controlling for age, revealed statistically significant time effects for several physiological parameters. Specifically, significant effects of time were observed in SMI (F(2, 28) = 1.94, p = 0.162, η² = 0.026), ECW/TBW (F(2, 28) = 4.34, p = 0.023, η² = 0.094), phase angle (F(2, 28) = 3.73, p = 0.035, η² = 0.085), LDL-C (F(2, 28) = 6.98, p = 0.004, η² = 0.143), and HbA1c (F(2, 28) = 5.21, p = 0.012, η² = 0.103). There were significant changes in SMI, ECW/TBW, phase angle, LDL-C, and HbA1c following the intervention, while other parameters showed minimal or no significant variation by post-hoc comparisons using paired Student's t-tests and Wilcoxon signed-rank tests, depending on data distribution (Table 3).

**Table 3:** Pairwise Comparisons of Physiological Parameters Between Three Measurement Periods in Females *Statistical significance was set at p < 0.05. Hₐ μ Measure 1 − Measure 2 ≠ 0. Normality testing via Shapiro–Wilk tests. t, Student's t and Cohen's d for test comparisons; W, Wilcoxon W and rank biserial correlation for test comparisons. Data are presented as mean ± SE or median (interquartile range), depending on data distribution. Test time points: 1, baseline (before the pre-intervention period); 2, pre (after the two-month pre-intervention period); 3, post (after the two-month intervention period). df, degrees of freedom; ECW/TBW, extracellular-to-total body water ratio; HbA1c, hemoglobin A1c; HDL-C, high-density lipoprotein cholesterol; HOMA-IR, homeostatic model assessment for insulin resistance; IRI, immunoreactive insulin; LDL-C, low-density lipoprotein cholesterol; QUICKI, quantitative insulin sensitivity check index; SE, standard error; SMI, skeletal muscle mass index; TC, total cholesterol; TG, triglycerides

Comparisons of Periods			Difference		95% Confidence Interval		Effect Size	Student's t or Wilcoxon W			
			Mean	SE	Lower	Upper		Statistic		df	p-value
fat_mass_2	vs.	fat_mass_1	-0.12	0.26	-0.68	0.44	-0.11	t	-0.46	15	0.656
fat_mass_3	vs.	fat_mass_1	-0.34	0.35	-1.08	0.40	-0.24	t	-0.97	15	0.348
fat_mass_3	vs.	fat_mass_2	-0.22	0.21	-0.67	0.23	-0.26	t	-1.03	15	0.318
muscle_mass_2	vs.	muscle_mass_1	-0.53	0.33	-1.23	0.18	-0.39	t	-1.58	15	0.136
muscle_mass_3	vs.	muscle_mass_1	-0.19	0.34	-0.91	0.53	-0.14	t	-0.57	15	0.574
muscle_mass_3	vs.	muscle_mass_2	0.33	0.27	-0.23	0.90	0.31	t	1.25	15	0.230
SMI_2	vs.	SMI_1	-0.18	0.08	-0.34	-0.02	-0.60	t	-2.39	15	0.031*
SMI_3	vs.	SMI_1	-0.13	0.10	-0.34	0.08	-0.33	t	-1.31	15	0.209
SMI_3	vs.	SMI_2	0.05	0.07	-0.10	0.35	0.18	W	40.00		0.559
ECW/TBW_2	vs.	ECW/TBW_1	0.00	0.00	0.00	0.00	-0.15	t	-0.58	15	0.569
ECW/TBW_3	vs.	ECW/TBW_1	0.00	0.00	-0.01	0.00	-0.95	t	-3.79	15	0.002*
ECW/TBW_3	vs.	ECW/TBW_2	0.00	0.00	0.00	0.00	-1.04	t	-4.15	15	< .001*
phase_angle_2	vs.	phase_angle_1	-0.05	0.05	-0.16	0.06	-0.24	t	-0.94	15	0.362
phase_angle_3	vs.	phase_angle_1	0.20	0.08	0.10	0.40	0.73	W	78.50		0.023*
phase_angle_3	vs.	phase_angle_2	0.21	0.07	0.07	0.35	0.82	t	3.26	15	0.005*
LDL-C_2	vs.	LDL-C_1	2.00	8.77	-13.00	17.00	0.10	W	66.00		0.755
LDL-C_3	vs.	LDL-C_1	-10.50	5.78	-24.00	-1.00	-0.65	W	24.00		0.024*
LDL-C_3	vs.	LDL-C_2	-16.00	5.99	-29.50	-8.00	-1.00	W	0.00		0.001*
HDL-C_2	vs.	HDL-C_1	-1.13	3.21	-7.97	5.72	-0.09	t	-0.35	15	0.731
HDL-C_3	vs.	HDL-C_1	-7.11	3.91	-15.44	1.21	-0.46	t	-1.82	15	0.089
HDL-C_3	vs.	HDL-C_2	-5.99	2.95	-12.28	0.30	-0.51	t	-2.03	15	0.061
T-Cho_1	vs.	T-Cho_2	-5.31	25.30	-54.90	44.28	-0.10	t	-0.66	15	0.520
T-Cho_1	vs.	T-Cho_3	-5.00	24.64	-53.29	43.29	-0.10	t	-1.05	15	0.310
T-Cho_2	vs.	T-Cho_3	0.31	24.45	-47.60	48.23	0.01	t	0.05	15	0.963
TG_2	vs.	TG_1	8.75	8.67	-9.72	27.20	0.25	t	1.01	15	0.329
TG_3	vs.	TG_1	6.50	9.16	-13.03	26.00	0.18	t	0.71	15	0.489
TG_3	vs.	TG_2	-8.00	11.21	-30.00	19.00	-0.32	t	35.50		0.300
HbA1c_2	vs.	HbA1c_1	-0.01	0.07	-0.16	0.14	-0.02	t	-0.09	15	0.930
HbA1c_3	vs.	HbA1c_1	0.20	0.04	0.05	0.30	0.78	W	49.00		0.032*
HbA1c_3	vs.	HbA1c_2	0.12	0.07	-0.03	0.27	0.41	t	1.65	15	0.120
IRI_2	vs.	IRI_1	-0.47	0.85	-2.28	1.34	-0.14	t	-0.55	15	0.589
IRI_3	vs.	IRI_1	1.13	1.28	-0.70	4.65	0.32	t	90.00		0.266
IRI_3	vs.	IRI_2	1.40	1.67	-0.70	4.75	0.35	t	92.00		0.224
HOMA-IR_2	vs.	HOMA-IR_1	-0.11	0.18	-0.50	0.28	-0.15	t	-0.60	15	0.558
HOMA-IR_3	vs.	HOMA-IR_1	0.27	0.32	-0.16	1.33	0.43	W	97.00		0.144
HOMA-IR_3	vs.	HOMA-IR_2	0.37	0.40	-0.04	1.22	0.46	W	99.00		0.117
QUICKI_2	vs.	QUICKI_1	0.00	0.01	-0.02	0.02	-0.04	t	-0.16	15	0.877
QUICKI_3	vs.	QUICKI_1	-0.02	0.01	-0.05	0.01	-0.46	W	37.00		0.114
QUICKI_3	vs.	QUICKI_2	-0.02	0.01	-0.04	0.00	-0.48	t	-1.90	15	0.076

The SMI decreased significantly between baseline and the pre-intervention test (p = 0.031, Cohen's d = −0.60), but there were no significant differences between other time points. The ECW/TBW ratio, reflecting hydration balance, decreased significantly from baseline to the post-intervention test (p = 0.002, Cohen's d = −0.95) and from the pre- to post-intervention tests (p < 0.001, Cohen's d = −1.04). These results suggest that hydration status improved during the intervention.

The phase angle, a marker of cellular health and membrane integrity, increased significantly between baseline and the post-intervention test (p = 0.023, Cohen's d = 0.73) and between the pre- and post-intervention tests (p = 0.005, Cohen's d = 0.82), indicating improved cellular function. LDL-C levels decreased significantly from baseline to the post-intervention test (p = 0.024, Cohen's d = −0.65) and from the pre- to post-intervention test (p = 0.001, Cohen's d = −1.00), suggesting improvements in lipid metabolism. While HDL-C levels decreased, these changes were not statistically significant.

The TC levels remained stable, with no significant differences between any of the time periods. Similarly, fat mass, muscle mass, and insulin-related indices such as HOMA-IR and QUICKI did not change significantly. However, HbA1c levels increased significantly from baseline to the post-intervention test (p = 0.032, Cohen's d = 0.78), suggesting a modest elevation in glycemic control markers.

Physiological and metabolic effects of the intervention in men

In male participants, repeated-measures ANOVA with age as a covariate demonstrated a significant main effect of time for LDL-C only (F(2, 22) = 6.98, p = 0.004, η² = 0.032). No significant time effects were found for muscle mass (F(2, 22) = 1.02, p = 0.377, η² = 0.007), SMI (F(2, 22) = 1.16, p = 0.333, η² = 0.021), or HDL-C (F(2, 22) = 2.37, p = 0.117, η² = 0.024). However, subsequent post-hoc tests (Table 4) identified several significant pairwise differences.

**Table 4:** Pairwise Comparisons of Physiological Parameters Between Three Measurement Periods in Males *Statistical significance was set at p < 0.05. Hₐ μ Measure 1 − Measure 2 ≠ 0. Normality testing via Shapiro–Wilk tests. t; Student t and Cohen's d for period comparisons, W; Wilcoxon W and rank biserial correlation for period comparisons. Test time points: 1, baseline (before the pre-intervention period); 2, pre (after the two-month pre-intervention period); 3, post (after the two-month intervention period). Data are presented as mean ± SE or median (interquartile range), depending on data distribution. df, degrees of freedom; ECW/TBW, extracellular-to-total body water ratio; HbA1c, hemoglobin A1c; HDL-C, high-density lipoprotein cholesterol; HOMA-IR, homeostatic model assessment for insulin resistance; IRI, immunoreactive insulin; LDL-C, low-density lipoprotein cholesterol; QUICKI, quantitative insulin sensitivity check index; SE, standard error; SMI, skeletal muscle mass index; TC, total cholesterol; TG, triglycerides

Comparisons of Periods			Difference		95% Confidence Interval		Effect Size	Student's t or Wilcoxon W			
			Mean	SE	Lower	Upper		Statistic		df	p-value
fat_mass_2	vs.	fat_mass_1	0.15	0.66	-0.70	1.10	0.09	W	49.50		0.807
fat_mass_3	vs.	fat_mass_1	-1.02	0.48	-2.06	0.03	-0.59	t	-2.12	12	0.056
fat_mass_3	vs.	fat_mass_2	-0.50	0.88	-2.45	0.05	-0.57	W	19.50		0.075
muscle_mass_2	vs.	muscle_mass_1	0.15	0.76	-1.75	1.40	0.05	W	41.00		0.906
muscle_mass_3	vs.	muscle_mass_1	1.67	0.53	0.52	2.82	0.88	t	3.17	12	0.008*
muscle_mass_3	vs.	muscle_mass_2	1.30	0.97	-0.05	3.50	0.60	W	73.00		0.059
SMI_2	vs.	SMI_1	-0.05	0.12	-0.30	0.45	-0.20	W	22.00		0.610
SMI_3	vs.	SMI_1	-0.23	0.09	-0.42	-0.04	-0.72	t	-2.60	12	0.023*
SMI_3	vs.	SMI_2	-0.20	0.13	-0.75	0.05	-0.64	W	10.00		0.083
ECW/TBW_2	vs.	ECW/TBW_1	0.00	0.00	0.00	0.00	-0.52	t	-1.86	12	0.088
ECW/TBW_3	vs.	ECW/TBW_1	0.00	0.00	0.00	0.00	-0.03	t	-0.10	12	0.926
ECW/TBW_3	vs.	ECW/TBW_2	0.00	0.00	0.00	0.01	0.15	W	38.00		0.688
phase_angle_2	vs.	phase_angle_1	0.05	0.16	-0.20	0.45	0.18	W	46.00		0.608
phase_angle_3	vs.	phase_angle_1	-0.06	0.16	-0.41	0.29	-0.11	t	-0.39	12	0.706
phase_angle_3	vs.	phase_angle_2	-0.08	0.16	-0.70	0.20	-0.24	W	25.00		0.503
LDL-C_2	vs.	LDL-C_1	3.95	3.57	-3.83	11.73	0.31	t	1.11	12	0.291
LDL-C_3	vs.	LDL-C_1	-12.72	2.93	-19.11	-6.33	-1.20	t	-4.34	12	< .001*
LDL-C_3	vs.	LDL-C_2	-16.67	2.59	-22.31	-11.03	-1.79	t	-6.44	12	< .001*
HDL-C_2	vs.	HDL-C_1	0.70	2.54	-4.40	4.60	0.13	W	51.50		0.701
HDL-C_3	vs.	HDL-C_1	-6.89	3.05	-13.54	-0.25	-0.63	t	-2.26	12	0.043*
HDL-C_3	vs.	HDL-C_2	-6.05	2.18	-10.79	-1.31	-0.77	t	-2.78	12	0.017*
T-Cho_1	vs.	T-Cho_2	-5.77	15.57	-36.28	24.74	-0.20	t	-1.26	15	0.232
T-Cho_1	vs.	T-Cho_3	-1.00	15.14	-30.68	28.68	-0.04	t	-0.26	15	0.796
T-Cho_2	vs.	T-Cho_3	4.77	16.66	-27.89	37.43	0.16	t	1.22	15	0.245
TG_2	vs.	TG_1	-3.00	16.83	-21.50	37.00	-0.05	W	43.00		0.893
TG_3	vs.	TG_1	12.62	8.45	-5.79	31.00	0.41	t	1.49	12	0.161
TG_3	vs.	TG_2	7.00	17.67	-22.00	37.00	0.20	W	54.50		0.552
HbA1c_2	vs.	HbA1c_1	-0.04	0.04	-0.13	0.06	-0.25	t	-0.89	12	0.391
HbA1c_3	vs.	HbA1c_1	-0.10	0.04	-0.30	0.00	-0.64	W	5.00		0.145
HbA1c_3	vs.	HbA1c_2	-0.03	0.06	-0.16	0.09	-0.15	t	-0.54	12	0.600
IRI_2	vs.	IRI_1	1.18	0.81	-0.60	2.95	0.40	t	1.45	12	0.174
IRI_3	vs.	IRI_1	0.69	0.72	-0.88	2.26	0.27	t	0.96	12	0.355
IRI_3	vs.	IRI_2	-0.49	0.88	-2.40	1.43	-0.15	t	-0.55	12	0.592
HOMA-IR_2	vs.	HOMA-IR_1	0.26	0.21	-0.18	0.71	0.36	t	1.28	12	0.224
HOMA-IR_3	vs.	HOMA-IR_1	0.14	0.17	-0.23	0.51	0.23	t	0.82	12	0.428
HOMA-IR_3	vs.	HOMA-IR_2	-0.13	0.20	-0.56	0.31	-0.18	t	-0.63	12	0.540
QUICKI_2	vs.	QUICKI_1	-0.02	0.01	-0.05	0.01	-0.31	t	-1.12	12	0.287
QUICKI_3	vs.	QUICKI_1	-0.01	0.01	-0.04	0.02	-0.25	t	-0.88	12	0.394
QUICKI_3	vs.	QUICKI_2	0.00	0.01	-0.02	0.03	0.09	t	0.32	12	0.755

Muscle mass increased significantly from baseline to the post-intervention test (p = 0.008, Cohen's d = 0.88), reflecting a notable improvement in muscular health. Similarly, the SMI decreased significantly from baseline to the post-intervention test (p = 0.023, Cohen's d = −0.72), indicating slight changes in skeletal muscle quality over the intervention period. However, there were no significant differences across the other time points for muscle mass or SMI.

The LDL-C levels in men decreased significantly between baseline and the post-intervention test (p < 0.001, Cohen's d = −1.20) and between the pre- and post-intervention tests (p < 0.001, Cohen's d = −1.79). These results suggest improvements in lipid metabolism. HDL-C levels also declined significantly between baseline and the post-intervention test (p = 0.043, Cohen's d = −0.63) and between the pre- and post-intervention tests (p = 0.017, Cohen's d = −0.77).

There were no significant changes in TC or TG across the study period. Similarly, the markers of glycemic control (HbA1c, IRI, HOMA-IR, and QUICKI) remained stable throughout the intervention.

## Discussion

This study demonstrated that the intake of cumin powder over two months produced different effects on body composition and lipid profiles in men and women. In the body composition analysis, women showed improved hydration status, as indicated by changes in the ECW/TBW ratio, and stabilization of cell membrane function, as evidenced by a significant increase in phase angle, suggesting a positive effect on muscle quality. However, in men, the impact on muscle quality was limited. We also observed some gender differences in the lipid profiles. In both men and women, LDL-C decreased significantly, while there was a decrease in HDL-C in men only. The other lipid and blood glucose profiles were stable or only slightly affected in both groups. These results suggest that cumin powder exerts its health effects in a gender-dependent manner, indicating the potential for personalized spice use tailored to the individual conditions and constitutions of each sex.

The BIA device used in this study is a well-established method for assessing musculoskeletal composition and is capable of evaluating both muscle mass and muscle quality [14,27,28]. While previous studies have investigated the effects of spices, including cumin, on muscle mass and body fat percentage, little attention has been given to their impact on muscle quality. One randomized controlled trial involving overweight or obese women reported that consuming 3 g/day of cumin powder with yogurt over three months significantly reduced body fat mass and percentage [6]. In contrast, our study did not observe significant changes in fat or muscle mass. This discrepancy may be due to differences in participant health status (our cohort consisted of healthy adults) and a shorter intervention period.

Notably, we found significant improvements in muscle quality indicators, specifically in women. ECW/TBW significantly decreased, and phase angle significantly increased, from pre- to post-intervention in the female subgroup. These changes were not observed in men, indicating a sex-specific response to cumin supplementation. The reduction in ECW/TBW suggests improved cellular hydration and reduced extracellular water retention, while the increase in phase angle reflects enhanced cell membrane integrity and overall muscle quality.

This sexual dimorphism likely results from interactions between cumin's phytochemical components and sex-specific physiological pathways. Apigenin, a predominant flavonoid in cumin [29], has demonstrated myotrophic effects in animal models by activating Akt/mTOR signaling, promoting skeletal muscle hypertrophy, and preventing atrophy in obese mice [30,31]. These effects may manifest more strongly in women due to the weak phytoestrogenic activity of apigenin and luteolin [29,32,33], which could augment estrogen receptor-mediated pathways that contribute to muscle preservation and quality enhancement. The absence of change in men may be attributable to the lack of these synergistic hormonal effects.

Regarding the effects of cumin on lipid profiles, a meta-analysis revealed that it is associated with significant reductions in TC and LDL-C levels and an increase in HDL-C levels [34]. In the present study, we observed no significant changes in TC or TG levels, but LDL-C levels decreased significantly in both men and women, consistent with previous research [34]. The mechanism behind the reduction in LDL-C may involve the flavonoids in cumin, which enhance paraoxonase-1 levels, and trace elements such as iron, zinc, and manganese, which increase the activity of antioxidant enzymes like superoxide dismutase. Components like saponins may also inhibit intestinal cholesterol absorption and promote its excretion; furthermore, cumin has been reported to upregulate LDL receptor expression, contributing to LDL-C reduction [34]. In contrast to previous studies, however, we observed a significant decrease in HDL-C in men and a nonsignificant decrease in women [34]. One possible explanation for this sex difference is the influence of estrogen. One estrogen, estradiol, is known to reduce LDL-C and increase HDL-C levels [35]. Our study group included relatively young women, suggesting that the effects of estradiol may have mitigated the decrease in HDL-C compared to men.

Although there are differences in results among studies regarding blood glucose profiles, a systematic review and meta-analysis reported no significant effects on indicators such as blood glucose levels, insulin secretion, and insulin resistance, which is consistent with our findings [36]. We also obtained no clear findings regarding sex differences.

Cumin has traditionally been used to treat not only lifestyle-related diseases but also effects specific to women, such as promoting menstruation and enhancing lactation [14]. In this study, its effects were more limited in men than in women, suggesting that active consumption is recommended for women. In the future, further research is needed on food intake and the use of spices to improve health, with a focus on sex differences.

Our study observed no adverse effects among participants during the two-month intervention period, which aligns with the established safety profile of cumin in previous clinical trials [34]. The literature indicates potential allergic reactions in individuals with sensitivities to plants in the Apiaceae family; however, our screening process effectively excluded such individuals. The administered dosage of 2 g/day was well-tolerated by all participants in our study population of healthy adults.

In contrast to previous studies that used higher daily doses of cumin (e.g., 3 g/day) and often targeted populations with metabolic disorders such as obesity or dyslipidemia, our study employed a moderate dose of 2 g/day in a healthy adult population. This lower dosage and the absence of pre-existing metabolic abnormalities may explain why we did not observe significant changes in fat mass, TC, or glucose metabolism. These findings suggest that the physiological effects of cumin may be dose-dependent and influenced by baseline health status. Future studies are needed to explore optimal dosage thresholds and to identify populations that may benefit most from cumin supplementation.

In interpreting these results, the statistical strategy employed deserves consideration. Given the known physiological and metabolic differences between men and women, we deliberately chose not to conduct a two-way repeated-measures ANOVA with sex as a between-subject factor. Instead, we treated sex as a biological stratification factor and performed repeated measures analyses separately within each sex group. This approach aligns with the study's exploratory objective to examine sex-specific responses to cumin intake, rather than to statistically compare sexes. Moreover, while omnibus repeated-measures ANOVA did not reveal significant main effects in some variables, we conducted pairwise comparisons across time points using appropriate tests (i.e., paired t-tests or Wilcoxon signed-rank tests, depending on data distribution). These post-hoc tests were justified by the pilot nature of the study, which aimed to generate preliminary insights and identify trends that may inform future hypothesis-driven research. This analytical strategy enabled us to capture subtle time-dependent changes within each sex, which may have been masked in an interaction model due to limited sample size and statistical power.

Limitations

This study was a pilot trial conducted on healthy Japanese adults without any prior medical history. Several limitations should be acknowledged, including the small sample size, the lack of randomization, and the absence of an appropriate control group. The female participants covered a broad age range (23-63 years), likely including both premenopausal and postmenopausal individuals. Since the estrogenic effects of phytoestrogens such as apigenin and luteolin may differ based on menopausal status, caution is warranted in interpreting cumin's influence on muscle quality and lipid metabolism. Future studies should stratify female participants according to menopausal status to better capture potential hormonal interactions.

Furthermore, we were unable to evaluate participants' lifestyle habits in detail, and the method of cumin intake (e.g., preparation method or timing of consumption) was not standardized. The wide variation in both age and BMI also poses a limitation. In particular, BMI was not used as a stratification variable in the current analysis, which may have masked differential effects of cumin in individuals with low versus high body fat. While our aim was to explore overall sex-specific responses in a general population, future studies with larger sample sizes should consider stratification by BMI category to clarify the role of body composition in modulating the effects of cumin.

Given these limitations, the findings should be interpreted with caution. Nevertheless, the pre-post design with a preliminary observation period offers valuable initial insights into the sex-specific effects of cumin supplementation. Future studies with more robust methodology and targeted subgroup analyses will be essential to confirm and expand upon these preliminary findings.

## Conclusions

We found that cumin produced different effects on body composition and lipid profiles in men and women. In terms of body composition, we observed a decrease in the intracellular water ratio and an increase in phase angle in women, suggesting improvements in muscle quality. For lipid profiles, we observed a reduction in LDL-C in both men and women but a decrease in HDL-C in men only. These results suggest that cumin exerts sex-specific health effects, indicating the potential for personalized spice consumption recommendations tailored to individual conditions and constitutions based on sex differences.
